# Case report: “Fur stole and turtleneck” and “halter-back” signs: an expanded wardrobe for dermatomyositis

**DOI:** 10.3389/fimmu.2024.1400575

**Published:** 2024-06-05

**Authors:** Jonathan D. Ho, Andrew T. M. Burton, Trimane McKenzie, Ciara Best, Andrea Clare-Lyn Shue, Stephanie Smith-Matthews, Kimone Fraser, Asana Anderson

**Affiliations:** ^1^ Division of Dermatology, Department of Medicine, The University of the West Indies, Mona, Kingston, Jamaica; ^2^ Department of Pathology, The University of the West Indies, Mona, Kingston, Jamaica; ^3^ Department of Medicine, The University of the West Indies, Mona, Kingston, Jamaica; ^4^ Division of Dermatology, Bustamante Hospital for Children, Kingston, Jamaica; ^5^ George Town Hospital, Cayman Island Health Services Authority, George Town, Cayman Islands

**Keywords:** dermatomyositis, autoimmune connective tissue disease, shawl sign, cutaneous, dermatologic, skin of color, bullous dermatomyositis

## Abstract

A diagnosis of dermatomyositis requires recognition of distinct patterns of skin disease in combination with, and sometimes without, muscle weakness. Often, a striking contrast between involved and uninvolved areas is observed. Familiar patterns include eyelid and midfacial eruptions, Gottron papules/sign, and upper back (shawl sign), central chest (V/open collar sign), and lateral thigh (holster sign) involvement. More recently, new specific antibody/phenotype-associated patterns have been reported. We describe a case series of two distinct patterns of skin involvement in six adult patients with both classical and amyopathic dermatomyositis. Three had paraneoplastic disease. All had intermediate to richly pigmented skin; five were of Afro-Caribbean and one was of Asian-Caribbean descent. Four were men, and two were women. Ages ranged from 41 to 89 years. All patients had concomitant hallmark signs (facial, hand, and/or trunk signs). Three were amyopathic. The first pattern involved a sharply demarcated, horizontally oriented hyperpigmented patch/thin plaque across the shoulders and upper chest, extending up the anterior neck. The second was the combination of the classical upper back shawl distribution with distinct mid-back sparing and diffuse involvement of the lower back. Named patterns help with the recognition of skin rashes in dermatomyositis. Based on the current lexicon describing items of apparel, we liken the first pattern to a “fur stole and turtleneck” sign and the latter to a “halter-back” or “reflected-shawl” sign. Biopsies revealed hyperkeratosis and interface dermatitis, often with epidermal atrophy, compatible with dermatomyositis. These patterns perhaps represent the coalescence of already well-described signs, photo-exacerbation, koebnerization, mechanical stretch, and other currently unclear factors contributing to patterning in dermatomyositis. Pattern distribution recognition is particularly valuable in individuals with richly pigmented skin who may lack typical violaceous erythema. The distinct demarcation led to the initial misdiagnosis of allergic contact dermatitis or other exogenous dermatitis in most of our patients. Further work involves evaluation of antibody phenotype and internal involvement associations. Limitations include lack of specific antibody panels and longitudinal follow-up data.

## Introduction

Dermatomyositis (DM) is characterized by a combination of distinctly patterned cutaneous signs with or without associated muscle disease. Classical (hallmark) patterning includes Gottron papules/sign, heliotrope, V-neck, shawl, sleeve, and holster signs (1). More recently, specific antibody-associated reproducible non-hallmark phenotypes have expanded the spectrum of patterned skin disease. Psoriasiform lesions, verrucous palmar papules, red-on-white lesions, and ovoid palatal patches have been described with anti-TIF1-γ antibodies while ulcerating, and inverse Gottron papules are seen in patients with antibodies against MDA5 (2–5). Although skin biopsies reveal characteristic interface dermatitis (ID), the light microscopic findings are indistinguishable from acute/subacute and occasionally chronic cutaneous lupus erythematosus (6). Recognition of the typical and expanding patterns of skin involvement is therefore crucial to arrive at the correct diagnosis, particularly in individuals with richly pigmented skin and amyopathic disease, cases where non-hallmark features predominate, or in resource-poor settings with limited access to ancillary antibody testing. We describe a series of two distinct patterns of skin involvement in six adult patients with both classical and amyopathic DM. Based on the current apparel-based lexicon describing trunk/proximal limb signs, we designate these signs “fur stole and turtleneck” sign (FSTNS) and “halter-back” sign (HBS).

## Case descriptions

### Case 1

**Figure 1:**
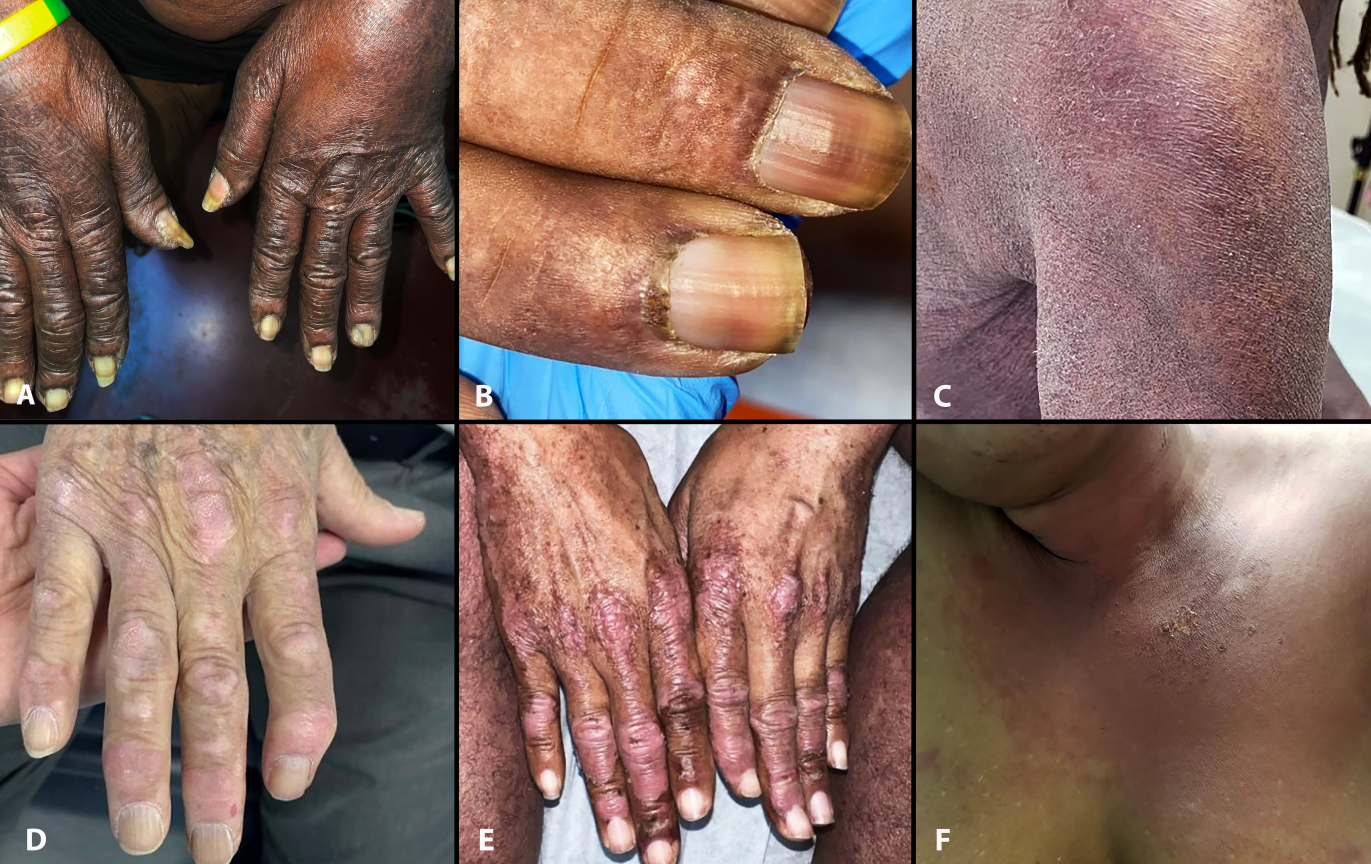
Select cutaneous manifestations of dermatomyositis in reported patients. Note Gottron sign/papules **(A)**, “ragged” cuticular hypertrophy in Case 2 **(B)**, and typical violaceous erythema, scale and “sleeve” sign in Case 3 **(C)**. Cases 1–3 also had a fur stole and turtleneck sign. Those with a halter-back/reflected shawl sign demonstrate Gottron papules (Cases 4 and 5, **D**, **E**) and V-sign (Case 6, **F**). Case 6 also had erosion and blisters (not shown).

**Figure 2:**
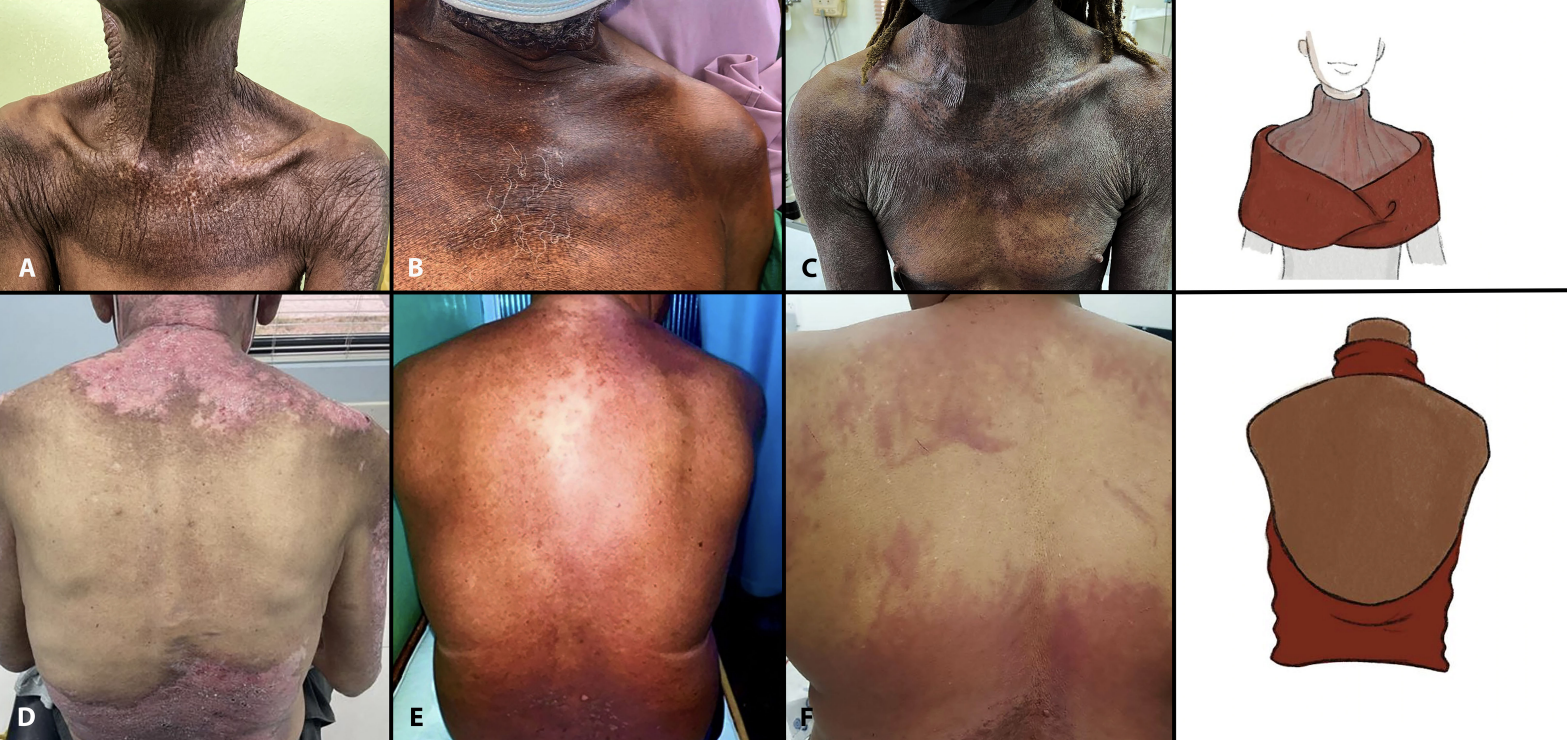
“Fur stole and turtle neck” and “Halter-back/reflected shawl” signs. Note the horizontally oriented sharply demarcated involvement of the upper chest and anterior shoulders (fur stole pattern) with diffuse involvement of the anterior neck (turtleneck pattern) in Cases 1–3 **(A–C)**. Observe the varied primary morphologies including atrophic hyperpigmentation with focal depigmentation **(A)**, macular hyperpigmentation (Case 2, **B**), and thin hyperkeratotic, hyperpigmented, and violaceous plaques (Case 3, **C**). Halter-back/reflected shawl sign (Cases 4–6, **D–F**) exhibits upper and lower back involvement with distinct sparing of the mid-back. Again, note the variable primary morphologies including psoriasiform plaques (Case 4, **D**), macular hyperpigmentation/red-brown erythema (Case 5, **E**), and macular diffuse and flagellate erythema (Case 6, **F**). This latter patient has antibodies against NXP-2 and SSA 52 kd.

**Figure 3:**
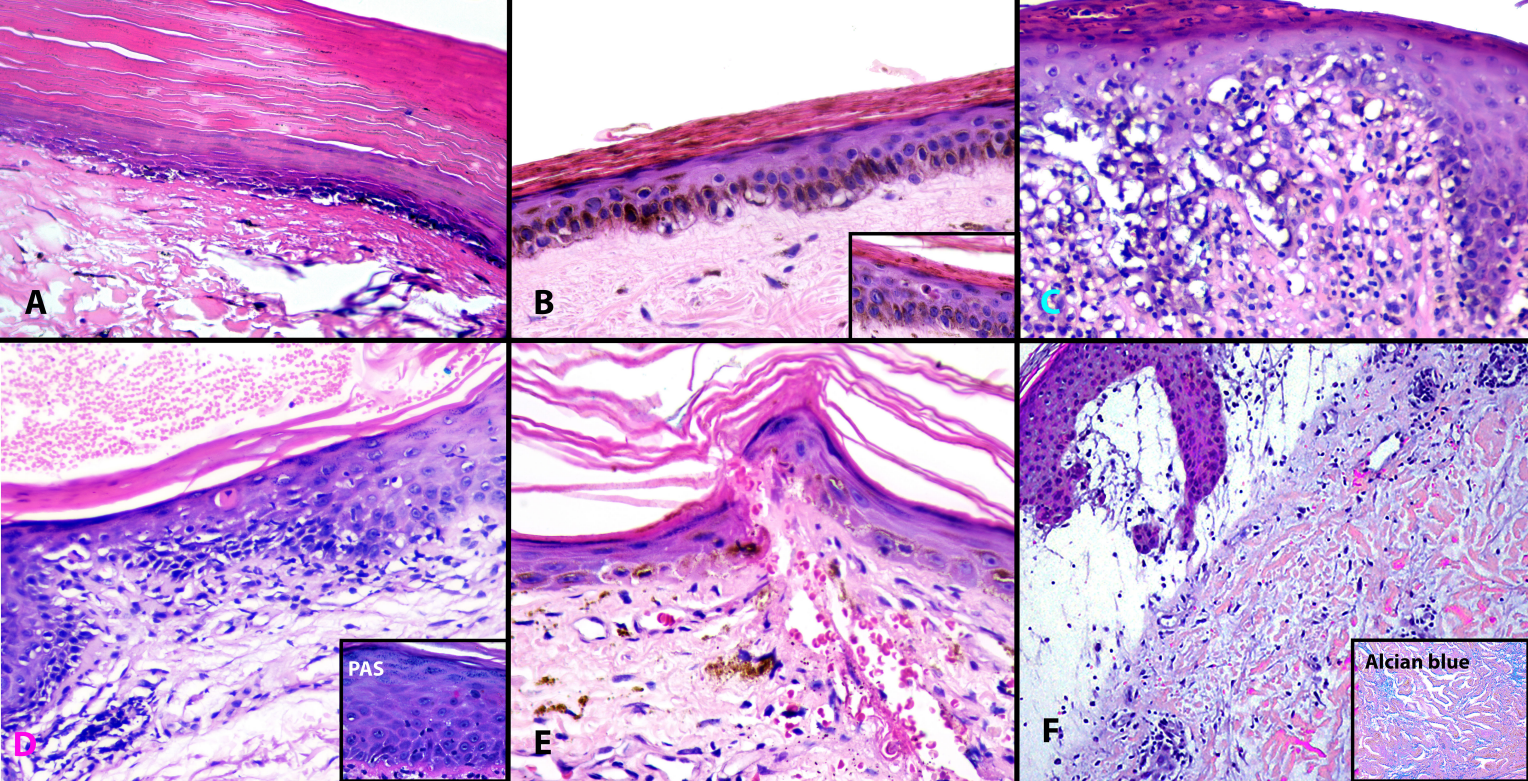
Histopathologic findings in “Fur stole and turtle neck” and “Halter-back/reflected shawl” signs. All cases demonstrated interface dermatitis. Note the hyperkeratosis, epidermal atrophy, and basal vacuolar change in biopsies from Cases 1, 2, and 4 (**A, B**, **D**). Apoptotic keratinocytes are visualized (**B** inset, **D**, **E**). Case 3 **(C)** has mild epidermal hyperplasia and a more prominent lymphocytic infiltrate while Case 6 **(F)** demonstrates massive papillary dermal edema and increased dermal mucin, highlighted by Alcian blue (**F**, inset). **(A–E)**, Hematoxylin and eosin (H&E) ×200; D inset, Periodic acid Schiff (PAS) ×400; F, H&E ×100; F inset, Alcian Blue ×200.

An 80-year-old Afro-Caribbean woman presented to the dermatology clinic for evaluation of a hyperpigmented and pruritic eruption on the chest, present for 3 years. She had a history of Hashimoto thyroiditis, hypertension, and gastroesophageal reflux disease maintained on levothyroxine, nifedipine, and lansoprazole. Recent blood work revealed a low-titer positive ANA (1:40) and positive anti-centromere and anti-U1RNP antibodies. Anti-Ro/La/Jo-1/Sm/Scl-70 and ds-DNA were negative. She denied features of Raynaud phenomenon or skin tightening. She denied muscle weakness. She had been referred to exclude systemic sclerosis (SSc) or allergic contact dermatitis. The patient was unsure if her rash was photo-exacerbated. Physical examination revealed dark brown, scalded-appearing hyperpigmentation involving the eyelids, malar cheeks, external ear, and lateral thighs. Hyperpigmented patches and plaques overlying the joints and extending along the interarticular surface (Gottron papules with ray sign) were noted (**Figure 1A**). A hyperpigmented and hyperkeratotic patch with subtle background erythema on the upper back consistent with a shawl sign was present. Although pruritic, she denied pre-existing erythema. Dermoscopy revealed nail fold telangiectasia with drop-out capillary loops. The skin, while hyperpigmented and hyperkeratotic, was not sclerotic. No sclerodactyly, facial telangiectasia, or facial skin tightening was identified. The cutaneous findings were compatible with DM. Strikingly, sharply demarcated horizontally oriented hyperpigmentation with prominent skin markings and focal hypopigmentation was present across the anterior shoulders and upper chest and extending up the anterior neck (**Figure 2A**). There was distinct sparing of the lower chest. The pattern across the chest was reminiscent of a fur stole, while the confluent involvement of the neck resembled a turtleneck sweater. A skin biopsy of the anterior shoulder revealed hyperkeratosis, epidermal atrophy, basal layer vacuolation, squamatization of the basal layer, and melanophages consistent with an ID of autoimmune connective tissue disease (AICTD). Alcian blue revealed mildly increased dermal mucin (**Figure 3A**). No significant perivascular lymphocytic inflammation was present. Homogenization and sclerosis compatible with scleroderma were not present. Musculoskeletal examination revealed normal power. Creatinine kinase (CK) levels were normal (117 U/L). The patient was started on hydroxychloroquine sulfate 200 mg daily, prednisone 15 mg daily (tapered to 7.5 mg), mycophenolate mofetil 500 mg twice daily, and 0.05% clobetasol propionate ointment. Investigations including pulmonary function tests, CT-CAP, upper/lower gastrointestinal endoscopy, mammography, serum protein electrophoresis, serum CA-125, and CEA were recommended. Myositis antibody panel was also suggested but declined due to financial constraints. CT-CAP revealed changes compatible with interstitial lung disease. At the time of writing (1 year after diagnosis), no cancer has been detected and the skin disease is controlled.

### Case 2

A 73-year-old Afro-Caribbean man admitted for aspiration pneumonia with a history of hypertension (maintained on telmisartan and nifedipine) and dysphagia was referred to the dermatology in-patient service for evaluation of a hyperpigmented rash present for 3 months. The patient had concomitant proximal muscle weakness with a recent inability to ambulate. He also reported an 8-month history of generalized joint pain. He had a history of positive rheumatoid factor [RF, 106 IU/L (normal <40 IU/L)], anti-cyclic citrullinated peptide, and ANA (no titer available). Anti-Ro/La/Jo-1/Sm/Scl-70 and ds-DNA were negative. The patient was unsure if his rash was photo-exacerbated. Dermatologic examination revealed dark brown scalded-appearing hyperpigmentation involving the central face, eyelids, and external ear. A hyperkeratotic papule was seen overlying the right second metacarpal joint. Cuticular hypertrophy with intra-cuticular hemorrhage was present (**Figure 1B**). The features were consistent with DM. A sharply demarcated, horizontally oriented macular hyperpigmentation involving the upper chest, anterior shoulders, and anterior neck (fur stole and turtleneck sign) was noted (**Figure 2B**). He denied a pre-existing erythematous phase. A skin biopsy (anterior shoulder) revealed epidermal atrophy, subtle basal layer vacuolation apoptotic keratinocytes, little inflammation, and scattered heavily pigmented melanophages consistent with an ID (**Figure 3B**). Alcian blue was negative for dermal mucin. Musculoskeletal examination revealed reduced power (3/5) of the bilateral hip flexors. CK levels were elevated (1,575 U/L). Anti-Ro/La/Jo-1/Sm/Scl-70 and ds-DNA were negative. Additional investigations including muscle biopsy, malignancy screens, myositis antibodies, and pulmonary function tests were recommended but not performed due to financial constraints. The patient defaulted from follow-up.

### Case 3

A 60-year-old Afro-Caribbean man presented to the dermatology clinic for evaluation of a >1-year history of a pruritic rash, previously diagnosed as a contact/eczematous dermatitis. He had a history of schizophrenia but was not maintained on any medications. He denied muscle weakness but endorsed a history of recent decreased appetite and weight loss. The patient was unsure if his rash was photo-exacerbated. Dermatologic examination revealed violaceous erythema involving the malar cheeks and eyelids. Poikiloderma and scaly hyperpigmented plaques with background violaceous erythema were also seen in a shawl and sleeve-sign pattern (**Figure 1C**) and on the lateral abdomen. Similar lesions were also present in a sharply demarcated distribution involving the upper chest, anterior shoulders, and anterior neck (fur stole and turtle neck pattern, **Figure 2C**). Skin biopsy (chest) revealed hyperkeratosis, mild epidermal hyperplasia, basal layer vacuolation, apoptotic keratinocytes, a superficial perivascular and focally band-like lymphocytic infiltrate, and scattered melanophages consistent with an ID (**Figure 3C**). Alcian blue revealed increased dermal mucin. Shortly after biopsy, the patient developed erythematous plaques overlying the metacarpal and interphalangeal joints consistent with Gottron papules. The features were consistent with DM. Musculoskeletal examination revealed normal power and CK levels were normal (31 U/L). The patient was commenced on prednisone 60 mg daily, hydroxychloroquine sulfate 400 mg daily, and 0.05% clobetasol propionate ointment twice daily. ANA/Anti-Ro/La/Jo-1/Sm/Scl-70 and ds-DNA were negative. Myositis-antibody panel was not performed due to financial constraints. A computed tomography (CT) scan of the chest revealed a large necrotic mediastinal mass suspicious for malignancy. At the time of writing, the patient is awaiting biopsy of the mass.

### Case 4

An 89-year-old Asian-Caribbean man presented for evaluation of a pruritic rash present for 4 years and managed as chronic eczematous dermatitis. Dermatologic examination revealed violaceous erythema and hypopigmented patches involving the eyelids, photodistributed malar cheeks, forehead, anterolateral arms, and the V of the neck. Erythematous papules were present over the metacarpal and interphalangeal joints consistent with Gottron papules (**Figure 1D**). The findings were characteristic of DM. In addition, well-defined erythematous and psoriasiform plaques involving the upper (shawl sign) and lower back with distinct sparing of the mid-back was noted (**Figure 2D**). The distribution was reminiscent of a halter-back blouse or, given its mirror image, a “reflection” shawl sign. The spared skin was not limited to the inferior margin of the scapula. A biopsy (shoulder) revealed hyperkeratosis, epidermal atrophy, apoptotic keratinocytes, a sparse superficial perivascular lymphocytic infiltrate, increased dermal mucin, and a PAS-positive thickened basement membrane zone consistent with ID of AICTD (**Figure 3D**). He endorsed difficulty getting up from a seated position and muscle examination revealed decreased power at the hip flexors. While muscle enzymes were requested, the patient declined any bloodwork. He commenced 0.05% clobetasol propionate ointment, mycophenolate mofetil 1 g twice daily, and hydroxychloroquine sulfate 200 mg daily. CT scan of the pelvis revealed a bladder mass. Biopsy confirmed papillary urothelial carcinoma. Transurethral resection of bladder tumor resulted in marked improvement of skin disease.

### Case 5

A 54-year-old man presented with a 6-month history of a pruritic rash involving the trunk, scalp, and hands. He had a history of hypertension and dyslipidemia, maintained on nifedipine and simvastatin for >5 years. He also had a 3-year history of Stage IIIA erythrodermic mycosis fungoides, in remission and controlled on methotrexate 20 mg weekly and folic acid. He was previously maintained with narrow-band ultraviolet light therapy but had recently noted increased pruritus and redness after treatment and phototherapy was discontinued. Although initially interpreted as recurrent erythrodermic mycosis fungoides, the eruption evolved to violaceous erythema involving the scalp, erythematous lichenoid papules and plaques overlying the metacarpal and interphalangeal joints with interarticular extension (Gottron papules with Ray sign, **Figure 1E**), and mid-facial hyperpigmentation compatible with DM. Violaceous erythema was seen involving the upper and lower back, with striking sparing of the mid-back (halter-back/reflection shawl pattern, **Figure 2E**). The sparing was not limited to the inferior border of the scapula. Biopsies of the new rash (back and thigh) revealed hyperkeratosis, epidermal atrophy, apoptotic keratinocytes, a sparse superficial perivascular lymphocytic infiltrate, and prominent melanophages consistent with an ID (**Figure 3E**). No Pautrier microabscesses or lymphocytic atypia were seen to suggest mycosis fungoides. Dermal mucin was not increased. The patient had no weakness and a musculoskeletal examination revealed normal power. CK level was normal (134 U/L). Upper and lower GI endoscopy, scrotal ultrasound, CT-CAP, PSA levels, and serum protein electrophoresis were normal. Within 6 months, the patient developed night sweats, weight loss, and enlarged lymph nodes. Inguinal lymph node biopsy revealed an atypical lymphoid infiltrate with Reed–Sternberg cells. The infiltrate expressed CD30, CD15, and PAX-5 consistent with the nodal Hodgkin disease. Chemotherapy was commenced (adriamycin, bleomycin, vinblastine sulfate, and dacarbazine) with resolution of his DM-type rash.

### Case 6

A 51-year-old Afro-Caribbean woman with no history of chronic illness presented to the emergency room with a 2-month history of a pruritic skin rash diagnosed as an “allergic reaction” and prescribed antihistamines. She subsequently developed shoulder and thigh pain with progressive muscle weakness. Four days before presentation, she became unable to stand. Skin examination revealed diffuse erythema involving the upper chest (V-neck, **Figure 1F**), outer arms (sleeve sign), outer thighs (holster sign), and abdomen. Areas of detachment and both flaccid and tense bullae were noted. Striking flagellate and diffuse erythema were noted to involve the upper back and lower back with sparing of the mid-back (HBS/RSS, **Figure 2F**). Musculoskeletal examination revealed decreased power at the hips, elbows, and knees. Skin biopsy (arm) revealed basal layer vacuolar change, massive papillary dermal edema with subepidermal clefting, a sparse superficial perivascular lymphocytic infiltrate, and Alcian blue-positive increased dermal mucin (**Figure 3F**). The findings were consistent with DM. CK levels were elevated (3,866 U/L). A myositis antibody panel revealed positive anti-NXP-2 and anti-SSA 52 kd antibodies. The patient was commenced on prednisone 1 mg/kg and mycophenolate mofetil 1 g twice daily. Malignancy screens were recommended but none were performed. At the time of writing (20 months after diagnosis), the patient’s disease is well-controlled without known malignancy or internal disease.

Clinical/laboratory findings for Cases 1–6 are summarized in **Table 1**.

**Table 1:** Clinical and laboratory findings in patients with fur-stole, turtleneck, and halter-back/reflected shawl signs.

Details	Case 1	Case 2	Case 3	Case 4	Case 5	Case 6
**Demographics**	80 years old F	73 years old M	60 years old M	89 years old M	54 years old M	41 years old F
**Referral diagnosis**	Scleroderma Pellagra ACD	Dermatomyositis Scleroderma	Eczematous dermatitis Contact dermatitis	Pruritus Eczematous dermatitis	MF flare	ICD Allergic reaction
**Comorbidities**	Hashimoto thyroiditis HTN	None	Schizophrenia	HTN, DMII	MF HTN DM	None
**DM features**	Malar and eyelid pigmentation, GP, Ray sign, shawl/sleeve/holster signs, poikiloderma	Malar and eyelid pigmentation, GP, cuticular dystrophy, sleeve sign	Malar and eyelid erythema Shawl/sleeve signs, poikiloderma	Malar and eyelid erythema, GP, shawl, sleeve, V-sign	Mid-facial pigmentation, GP, Ray sign Shawl sign Violaceous scalp erythema	V-sign, sleeve sign Flagellate erythema Bullae
**Specific pattern**	FSTNS	FSTNS	FSTNS	HBS/RSS	HBS/RSS	HBS/RSS
**Biopsy**	Yes ID + EA	Yes ID + EA	Yes ID + PSEH	Yes ID + EA	Yes ID + EA	Yes ID + papillary dermal edema
**Autoantibody status**	ANA+, U1RNP+, centromere+	ANA+, RF+, CCP+	ANA− ENA−	NP	ANA− ENA−	Anti-NXP-2 and anti-SSA 52 kd
**Muscle status**	Amyopathic CK 117	Myopathic CK 1575	Amyopathic CK 31	Clinically myopathic CK NP	Amyopathic CK 134	Myopathic CK 3866
**Cancer associated**	Not at time of writing	Defaulted from care	Likely (large necrotic paratracheal mass, awaiting biopsy)	Yes (bladder papillary urothelial carcinoma	Yes Hodgkin lymphoma	Not at time of writing

ACD, allergic contact dermatitis; ANA, antinuclear antibody; CK, creatine kinase; CCP, cyclic citrullinated peptide; DMII, Type II diabetes mellitus; EA, epidermal atrophy; ENA, extractable nuclear antigens; FSTNS, fur stole and turtle neck sign; GP/S, Gottron papules; HBS, halter-back sign; HTN, hypertension; ID, interface dermatitis; ICD, irritant contact dermatitis; PSEH, psoriasiform epidermal hyperplasia; MF, mycosis fungoides; NP, not performed; RSS, reflected shawl sign.

## Discussion

We describe two striking and repeatedly encountered patterns of skin involvement in six adult patients with DM. We define FSTNS as a horizontally oriented eruption across the anterior shoulders and chest, extending up the anterior neck with sharp inferior demarcation from the lower chest and abdomen. It differs from the shawl sign (eruption over the upper back, neck, and posterior shoulders) and the V/open collar sign describing V-shaped erythema corresponding to the central chest (7). The HBS or “reflected-shawl” sign (RSS) is defined as upper (classical shawl) and mirror-image lower back involvement with distinct, horizontally oriented mid-back sparing. All forms, regardless of their primary morphology (see below), demonstrated characteristic ID, albeit with varying degrees of epidermal change and inflammation.

Regarding the clinical features of those with an FSTNS, all were Afro-Caribbean and had hallmark DM features. Case 1 had background autoimmunity (Hashimoto thyroiditis) and positive anti-U1RNP/centromere antibodies. Specifically, given her antibody status, we considered an overlap syndrome/MCTD with some features of SSc. We also note focal admixed hypopigmentation involving the chest. While admixed “salt-and-pepper” pigmentation of SSc cannot be entirely excluded, we consider most of her eruption a manifestation of DM for the following reasons: (1) While the patient is older and may have background age-related skin laxity, at the time of presentation, she continued to be symptomatic (pruritus) and therefore a “burnt-out” AICTD is unlikely. (2) Even in burnt-out SSc, biopsy findings should not have overlying ID, atrophy, or squamatization of the basal layer. (3) The patient’s malar, eyelid, sleeve-sign, articular, and shawl-sign pigmentation together with her chest eruption is highly patterned and sharply demarcated, features/distribution unusual in SSc. (4) While mixed hyper/hypopigmentation is most commonly seen in SSc, we recognize that it is not specific for this entity and similar findings have been reported in DM (8). The combination of epidermal atrophy and dyspigmentation favors poikiloderma of DM over SSc. Other entities presenting histologically with ID and post-inflammatory pigmentation, particularly lichen planus pigmentosus (LPP), could be entertained but distinct clinical features including often asymptomatic circular/oval patches on the trunk, diffuse (but non-patterned) pigmented patches with distinct blue-gray color, and predilection for the temporal and preauricular rather than malar face easily distinguish this condition (9). Case 2 had rheumatoid arthritis overlap. Two were amyopathic and one had muscle involvement. Case 1 has radiographic features suggestive of interstitial lung disease and Case 3 is likely malignancy-associated, although a definitive cancer diagnosis is pending. Importantly, as all are within 5 years of diagnosis, an associated malignancy/systemic disease may yet emerge in the remainder. Interestingly, while the distribution pattern was reproducible, the primary morphology varied. One patient had atrophic but hyperkeratotic and hyperpigmented skin, another had primarily macular hyperpigmentation, and the third had scale, background classical violaceous erythema, and admixed hyperpigmentation. None of the patients were suspected to have medication-induced disease.

Two of our patients with HBS/RSS were Afro-Caribbean and one was Asian-Caribbean. Two had muscle involvement and one was amyopathic. Similar to those with FSTNS, while the pattern of involvement was strikingly similar, the primary morphology was variable, including psoriasiform plaques, violaceous macular erythema, and diffuse and flagellate red macular erythema. Case 6 had the additional unique finding of vesiculobullous disease. While rare, bullous DM is well described and included as compatible in Sontheimer’s diagnostic criteria for ADM (10). Bullae are due to marked subepidermal edema as seen in our case (11). Very recently, like our patient, bullous DM has been described in a patent with anti-NXP2 antibodies (12).

Recognition of described patterns of cutaneous disease is crucial for early and accurate diagnosis of DM, highlighted by the initial misclassification in five of our six patients, three of whom had likely paraneoplastic disease. In particular, eczematous dermatitis was frequently considered, presumably because patterned itchy skin disease often generates a differential diagnosis of allergic contact dermatitis (13). Identification of distribution patterns is particularly important in individuals with richly pigmented skin where DM is often misclassified due to the frequent absence of morphologically typical violaceous erythema and predominance of pigmentary alteration (14). This is underscored in Cases 1 and 3 where pigmentation was the primary aberration and both patients denied noticeable precursor erythema. Although most dermatologists and rheumatologists are familiar with classical hallmark features, less familiar non-hallmark and atypical eruptions are increasingly described. Some of these have reproducible associations with specific antibodies including ovoid palatal patch/verrucous palmar papules/red-on-white patches with anti-TIF1-γ antibodies, calcinosis/edema with anti-NXP-2 antibodies, and inverse/ulcerated Gottron papules and anti-MDA5 antibodies (2–5, 15). Other non-classical/novel presentations without definitive/repeated antibody associations include whip-like or flagellate erythema (described in some individuals with anti-TIF1-γ antibodies but also in Case 6 with anti-NXP-2 disease), pityriasis rubra pilaris-like (Wong-type) DM, and perinasal swelling (16–18). We add FSTNS and HBS/SS to the recognizable patterns of skin disease in DM. Unfortunately, apart from Case 6, we are currently unable to link these patterns to specific autoantibodies.

What initiates patterned skin disease in DM? While sun exposure is the presumed culprit for some signs (malar erythema, sleeve sign, and V-sign), for lesions over bony prominences, trauma is a likely contributor. The development of unilateral Gottron papules on the functional hand of a patient with hemiparesis and inverse Gottron papules after participating in tug-of-war support a role for trauma (19, 20). Additionally, compared with photodistributed signs, Gottron papules exhibit mechanical stretch-related expression of CD44v7. Proposedly, in combination with abnormal amounts of osteopontin, there is a resulting increased inflammatory cell influx, raising the possibility that areas of increased physiologic skin stretch may induce cutaneous lesions of DM (21). While FSTNS could simply represent the coalescing of V and sleeve signs, unique characteristics include involvement of the anterior neck and the sharp inferior horizontal demarcation rather than the rounded apex of the V-sign. Perhaps a combination of photoexposure and well-established high skin tension of the anterior chest (22) contributes to this pattern, although involvement of the anterior neck remains unexplained by these and other proposed triggers having low skin tension, relative sun protection by the mandibular overhang, and not being particularly prone to trauma. For HBS/RSS, sparing of the mid-back is suggestive of the role of scratching contributing to this morphology. The pattern of central back sparing is strikingly similar to the “butterfly sign” seen in pruritic disorders, due to the inability to effectively reach, and thus pick/scratch, the central back, leading to post-inflammatory pigmentation or excoriations involving the upper and lower back but with uninvolved mid-back skin (23). As DM is not an exogenous or artifactual disease, scratching likely induces an isomorphic (koebnerized) response, inducing new disease in areas of induced trauma (24). In support, pruritus was reported in all our patients with this pattern. Like FSTNS, the posterior shoulders and upper back have increased skin tension (25). Skin tension compounded by physiologic movement around the shoulder joint and with bending could potentially contribute to this pattern. While these proposed mechanisms are plausible, we should also recall that certain variants of DM have unusual sparing/involvement patterns, unassociated with any of the factors described above, and we cannot exclude an unexplained predilection for our reported patterns. In particular, Wong-type DM, like its morphologic comparator disease, pityriasis rubra pilaris, has seemingly random islands of normal skin in a background of erythroderma (18). Additionally, an “angel-wing” pattern described as involvement of the upper and lower back, but with unusual sparing in an arched wing-like pattern along the inferior scapular border, has been described in a subset of Japanese patients with anti-SAE antibodies (26). While similar (and perhaps mechanistically related) to HBS/RSS, the sparing pattern was distinctly arched along the inferior border of the scapular rather than broad sparing of the mid-back. Although, based on the countries of origin of rare presentations, certain DM patterns may favor particular ethnic groups/geographic locations, at this time we are unable to determine if this is true for FSTNS and HBS/RSS. Nevertheless, these morphologic variants further highlight the tendency for distinct patterning of skin disease in DM and the need for clinician familiarity with the progressively protean presentations of this disease.

## Conclusion

In summary, we present two recognizable patterns of patterned skin disease in adult DM. Recognition should prompt early diagnosis and appropriate investigations. A sharply demarcated disease should not be viewed as unusual in DM. Although the distribution is strikingly similar, the primary morphology of the eruption varies. Biopsy demonstrates classical ID and will easily exclude exogenous causes of dermatitis including allergic contact etiologies. At this time, patients with FSTNS and HBS/RSS do not have a specific risk profile and should therefore be carefully evaluated for muscle disease, internal involvement, and associated malignancies. Further work includes evaluating for antibody–phenotypic associations. Limitations include a lack of confirmed myositis antibodies in the majority of patients and outstanding results, primarily due to patient financial constraints.

## Data availability statement

The original contributions presented in the study are included in the article/supplementary material. Further inquiries can be directed to the corresponding author.

## Ethics statement

Ethical approval was not required for the studies involving humans because case reports at our institution do not require IRB approval. The studies were conducted in accordance with the local legislation and institutional requirements. The participants provided their written informed consent to participate in this study. Written informed consent was obtained from the individual(s) for the publication of any potentially identifiable images or data included in this article.

## Author contributions

JH: Conceptualization, Data curation, Investigation, Methodology, Project administration, Resources, Supervision, Visualization, Writing – original draft, Writing – review & editing. AB: Data curation, Methodology, Resources, Writing – original draft, Writing – review & editing. TM: Data curation, Investigation, Methodology, Writing – original draft, Writing – review & editing. CB: Data curation, Methodology, Resources, Writing – original draft, Writing – review & editing, Investigation. AS: Data curation, Resources, Writing – original draft, Writing – review & editing. SS: Data curation, Investigation, Methodology, Resources, Writing – original draft, Writing – review & editing. KF: Data curation, Investigation, Resources, Writing – original draft, Writing – review & editing. AA: Conceptualization, Data curation, Investigation, Methodology, Writing – original draft, Writing – review & editing.
